# Practice Patterns of Graduates from a Surgical Oncology Fellowship Program

**DOI:** 10.1245/s10434-025-19073-z

**Published:** 2026-01-22

**Authors:** Aaron Scott, Andrew Nguyen, Christina Perez, Lawrence D. Wagman, Kurt Melstrom, Yuman Fong, Laleh Melstrom, Lily L. Lai

**Affiliations:** Division of Surgical Oncology, Department of Surgery, City of Hope, Duarte, CA USA

**Keywords:** Surgical oncology, Surgical education, Fellowship, Training, Surgery

## Abstract

**Background:**

Since its inception, surgical oncology training has emphasized the treatment of complex tumors without restriction to a specific anatomic region. As the complexity of cancer care increases, it is unclear whether this broad-based training model remains optimal. We set out to characterize how fellowship prepares graduates for clinical practice and to identify trends in surgical oncology training and practice.

**Methods:**

A 24-item survey was sent to graduates of an academic surgical oncology training program between the years 1981 and 2022. Questions focused on preparedness for practice upon graduation, current clinical and non-clinical activities, and the evolution of respondents’ practice since completion of fellowship.

**Results:**

Respondents indicated that they felt generally well prepared to treat a wide range of malignancies. Post-2013 graduates, felt significantly better prepared to treat peritoneal surface and gynecologic malignancies and to perform robotic surgery. In contrast, pre-2013 graduates indicated greater preparedness for the treatment of melanoma, extremity sarcoma, head and neck, esophageal and thoracic malignancies (p 0.05). Respondents practiced broadly, treating a median of 5 disease sites, although 36% dedicated more than half of their time to a single site. Despite the lack of formalized leadership training, 40.9% of graduates were involved in leadership positions.

**Conclusions:**

Our findings confirm that the existing surgical oncology training positions trainees well for practice, even in a landscape with many partially overlapping and competing surgical fellowships. In addition, many graduates are engaged in administrative and leadership roles suggesting that surgical oncologists may benefit from formalized leadership training during fellowship.

**Supplementary Information:**

The online version contains supplementary material available at 10.1245/s10434-025-19073-z.

Since its inception, surgical oncology training has emphasized multidisciplinary care and the treatment of rare tumors without restriction to a specific organ or anatomic region.^1^ Although training in surgical oncology dates back to at least the 1930s,^2^ it was not until 1981 that formalized guidelines were established under the auspices of the Society of Surgical Oncology (SSO) and the National Cancer Institute.^3^ As originally described, the surgical oncologist was expected to be a true surgical triple (or quadruple) threat. They were to be a broad-based surgeon treating cancer as well as a capable basic and clinical research scientist, an educator, and an institutional leader. Since 2011, surgical oncology has been recognized by the Accreditation Councils for Graduate Medical Education (ACGME) and the American Board of Surgery with subspecialty board certification.^4,5^

Cancer care, and indeed medicine in general, has changed significantly since the 1980s. However, the essential elements of surgical oncology training, as defined by Schweitzer et al.^3^ in 1981, continue as the basis for surgical oncology fellowship training. The degree to which the surgical oncology training paradigm reflects the reality of current surgical oncology practice remains unclear. For instance, radical neck dissection and hemipelvectomy were once considered routine cases of the surgical oncologist,^3^ but these operations are now largely performed by head and neck or orthopedic surgeons.^1^ Similarly, surgical oncologists may have been expected to “possess the ability to administer major regimens of non-operative therapy,” but the explosion of systemic therapies in the last several decades has rendered this infeasible. Defining the scope of surgical oncology is further confounded by the emergence of numerous overlapping surgical subspecialties. Thyroidectomies and parathyroidectomies are also performed by otolaryngologists, adrenalectomies by urologists, and extremity sarcomas are excised by orthopedic surgeons. Even within the specialty of general surgery, multiple subspecialty fellowships such as breast, colorectal, endocrine, hepatobiliary, and transplant surgery all overlap to some degree with surgical oncology. It has been noted that this identity confusion may contribute to declining interest in surgical oncology among graduating general surgery residents.^2,6^

In addition to clinical scope, other required components of surgical oncology training deserve reexamination. Research, long considered fundamental to the training and practice of surgical oncologists, has been a *de facto* requirement for matriculation into a surgical oncology fellowship^7^. Most surgical oncology fellows have completed 2 or more years of pre-fellowship research prior to fellowship training.^8^ With the increasing demands and sophistication of basic science that fuel the rapid pace of new discoveries, the required 4 months of dedicated research time in the ACGME Complex General Surgical Oncology (CGSO) fellowship curriculum may not be enough for those intent on an academic career. Furthermore, the decline in surgeon scientists, as documented and discussed in multiple publications, ^9,10^ also calls into question the research time as many surgical oncologists are not involved in research after their fellowship training.

Previous surveys have investigated the practice of surgical oncologists and have broadly supported the current training paradigm. Heslin et al.^11^ surveyed graduates from a single fellowship program between 1985 and 1996 and found that the majority of graduates were in academia and were highly satisfied with their jobs and that using broadly defined disease categories, fellowship training approximated clinical practice well.^11^ In a survey of CGSO graduates from 2005 to 2016, Ruff et al.^12^ found that most graduates practiced at an academic center and that most operated on a wide variety of malignancies. In another survey of CGSO graduates between 2012 and 2022, Behrens et al.^8^ respondents indicated feeling overall well prepared for practice, but areas of relative unpreparedness included research, thoracic surgery, hyperthermic intraperitoneal chemotherapy, and hepatopancreatobiliary surgery.

Using a cohort of graduates from a single surgical oncology fellowship between 1981 and 2022, we sought to characterize the current practice of fellowship graduates, to assess preparedness for practice upon graduation, and to determine the degree to which training aligned with practice. Taking advantage of the 40-year time period included in the survey, we sought to identify trends in training and practice and to assess the longitudinal evolution of the surgical oncology practice of the graduates. In so doing, we provide data that CGSO program directors can use to tailor their curriculum within the confines of the ACGME guidelines and that prospective surgical trainees can use to inform their choice of fellowship.

## Methods

Between 1981 and 2022, a total of 110 fellows completed advanced training in surgical oncology at City of Hope. Invitations to complete the electronic survey were given in person at the 2023 annual SSO meeting and via email using Survey Monkey to those for whom a valid email address was available.

The 24-item survey was developed with questions focusing on fellowship training and perceptions of readiness upon graduation, current practice, and evolution of practice in time (Supplementary Appendix A). All survey questions were developed by the authors (AS and LL). The initial set of questions was separately assessed for content validity by three additional surgeons, and the final survey included 24 questions. The survey took approximately 10 minutes to complete. No financial incentive was given.

Preparedness for practice was assessed across 15 oncologic disease sites: thyroid/parathyroid, other head and neck, breast, melanoma, thoracic (excluding esophagus), esophagus, stomach, pancreas, hepatobiliary, colon and rectum, peritoneal surface malignancy, retroperitoneal sarcoma, extremity sarcoma, gynecologic oncology, and urologic oncology. We also assessed preparedness for practice in the following non-oncologic or non-clinical areas: non-oncologic surgery in patients with cancer, basic science research, clinical research, public health, leadership/administration, and surgical education. For each area, respondents indicated their preparation for practice upon graduation on a scale from 1 to 5, where 1 indicated that the respondent felt inadequately prepared, 3 indicated that they felt adequately prepared, and 5 indicated that they felt very well prepared. Using the same areas, respondents were asked whether there were any areas they felt were under- or over-emphasized during training, with the additional option of a free response. Finally, using the same scale from 1 to 5, respondents indicated how well prepared they were to perform robotic surgery.

To allow us to assess current practice, graduates were asked to indicate the location of their current practice, practice setting (university/academic, academic affiliate, freestanding cancer center, private practice or other), years in practice, number of jobs since graduation, and number of partners. Respondents were then asked to estimate, in the last year, how much of the time was dedicated to the treatment of the previously listed oncologic disease sites and non-oncologic and non-clinical areas, with the addition of general surgery in patients without cancer. Percentages assigned to all areas were required to add up to 100%. Respondents were asked to indicate the degree to which they had integrated robotic surgery into their practice on a scale from 1 to 5. Finally, graduates were asked to indicate how the clinical breadth, research focus, time dedicated to leadership or administration, and time dedicated to education in their current position had changed since they took their first job. Six months after the initial survey, a 16-item follow-up survey was sent to those who had completed the first survey, with questions focusing on the respondents’ involvement and training in leadership/administration, surgical education, and robotic surgery (Supplementary Appendix B).

Respondents were divided into pre-2013 and post-2013 (including 2013) graduates, with the year 2013 corresponding to the first graduating class eligible for American Board of Surgery CGSO board certification. Variables were compared between these two groups using the Mann–Whitney U-test or Fisher’s exact test. Similar comparisons were performed between the current practice patterns of male and female respondents. All statistics were performed in IBM SPSS Statistics version 29 and Microsoft Excel.

## Results

A total of 92 survey invitations were sent and 51 responses were received (55% response rate). Response rates to individual questions varied from 86% (44/51) to 100%. There were 35 male and 16 female respondents. In the follow-up survey focusing on leadership, education, and robotic surgery, 51 emails were sent and 28 responses received (55% response rate). In total, 21 (41%) of respondents were board certified in CGSO; 28 (54%) respondents graduated before 2013 and 22 graduated in 2013 or later (43%). One respondent did not specify a year of graduation. Of the respondents who came into fellowship with a specific clinical interest, the most common answers were hepatopancreatobiliary (n=11), breast (n = 6), and peritoneal surface malignancy (n = 4).

Respondents were asked to rate their preparedness for practice at the time of graduation across several oncologic disease sites on a scale from 1 to 5, with a 1 representing inadequate preparation and 5 indicating that they felt very well prepared (Table 1). Respondents felt best prepared to treat malignancies of the colon and rectum, breast, melanoma, stomach, and pancreas, all of which had an average answer > 4. When we compared the responses from the pre-2013 graduates and those from the post-2013 graduates, the earlier cohort felt significantly better prepared to treat melanoma, stomach, endocrine, extremity sarcoma, head and neck, esophagus, and thoracic malignancies (all *p* < 0.05) than the later cohort. In contrast, the later cohort felt better prepared to treat peritoneal surface malignancies and gynecologic oncology (both *p* < 0.01). When asked about preparation for non-clinical activities, respondents felt generally well prepared, with the pre-2013 graduates indicating better preparation for basic science research (*p* = 0.02) and the post-2013 graduates feeling better prepared for leadership and public health (*p* = 0.026 and *p* = 0.043, respectively). The most common areas that respondents indicated they would have liked to have had more exposure to during fellowship were leadership/administration (n = 17), retroperitoneal sarcoma (n = 16), pancreas (n = 14), and hepatobiliary (n = 13). In contrast, the areas that were most frequently felt to be not useful or overemphasized during training were thoracic excluding esophagus (n = 9), gynecologic oncology (n = 7), head and neck other than endocrine (n = 5), and urologic oncology (n = 4).

**Table 1:** Average preparedness for practice at graduation as assessed by respondents on a scale from 1 to 5, with 1 indicating that respondents felt inadequately prepared, 3 indicating feeling adequately prepared, and 5 indicating feeling very well prepared

	Full cohort	Pre-2013 graduates	Post-2013 graduates	*p* value
Current clinical practice				
Colon and rectum	4.58	4.58	4.55	0.621
Breast	4.54	4.58	4.48	0.566
Melanoma	4.26	4.52	3.90	0.023
Stomach	4.22	4.42	3.95	0.020
Pancreas	4.20	4.21	4.25	0.897
Hepatobiliary	3.96	3.79	4.20	0.210
Retroperitoneal sarcoma	3.83	4.00	3.57	0.138
Thyroid/parathyroid	3.76	4.00	3.40	0.037
Non-oncologic surgery in cancer patients	3.76	3.76	3.70	0.849
Peritoneal surface malignancy	3.51	2.57	4.48	<0.001
Extremity sarcoma	3.50	4.17	2.58	<0.001
Other head and neck	2.87	3.44	2.00	<0.001
Esophagus	2.77	3.09	2.26	0.019
Gynecologic oncology	2.73	2.35	3.25	0.019
Thoracic oncology (excluding esophagus)	2.50	2.88	2.00	0.034
Urologic oncology	1.71	1.76	1.68	0.793
Non-clinical preparedness				
Clinical research	3.85	3.69	4.00	0.327
Surgical education	3.45	3.38	3.55	0.696
Leadership/administration	3.19	2.88	3.60	0.043
Basic science research	2.92	3.31	2.33	0.020
Public health research	2.72	2.36	3.15	0.026

Data are presented as averages unless otherwise indicated.

**Fig. 1:**
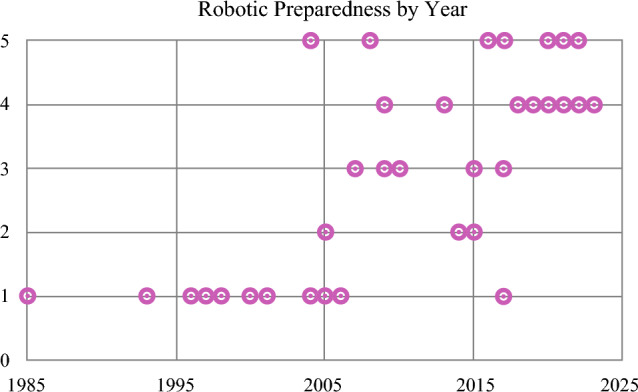
Preparedness to perform robotic surgery by year of graduation, with 1 indicating that respondents felt inadequately prepared, 3 indicating feeling adequately prepared and 5 indicating feeling very well prepared

Using the same scale from 1 to 5, the average response for preparedness to perform robotic surgery upon graduation across all years was 2.73 (Figure 1). However, there were significant differences across time. The average response prior to 2013 was 1.79, compared with 3.81 for the post-2013 graduates (*p* < 0.01). Prior to 2004, every respondent answered 1 (n = 11), indicating inadequate preparation to perform robotic surgery. There was a transitional period from 2004 to 2017 during which time the responses ranged from 1 to 5 (average 2.72, n = 22), and from 2018 onward all respondents indicated feeling well prepared, with answers ranging from 4 to 5 (average 4.38, n = 12).

Eight respondents had received subspecialty training other than surgical oncology: three before and 5 after fellowship. When asked whether they would still choose to complete a surgical oncology fellowship if given the opportunity to choose again, one respondent indicated that they would instead pursue alternative subspecialty training and one indicated that they would go into general surgery practice. The remaining 46 (96%) who answered the question would still choose surgical oncology.

**Fig. 2:**
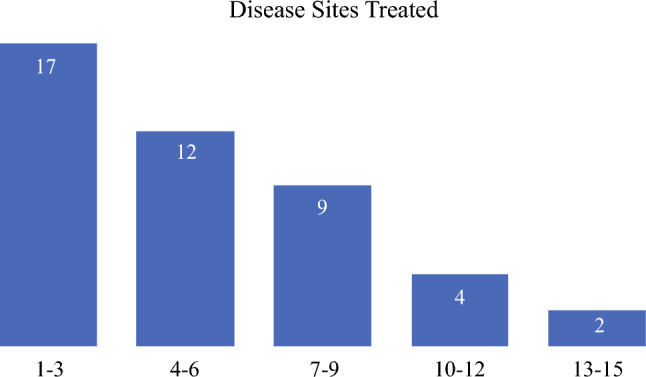
Number of different oncologic disease sites treated by respondents. The median number of disease sites treated was 5

The most common practice setting was in academics or at a university hospital (n = 23), followed by academic affiliate or hybrid practice (n = 10), private practice (n = 6), other (n = 4), and freestanding cancer center (n = 3). Respondents were geographically distributed widely, with California being the most common state to practice in. Respondents were asked to indicate, in the last year, what percentage of their time was spent treating several different disease sites or on non-clinical activities (Table 2). Based on 44 responses, the disease sites that respondents dedicated the most time to treating on average were melanoma, breast, pancreas, peritoneal surface malignancy, and colorectal. In contrast, the disease sites that respondents most frequently dedicated any amount of time to were melanoma, retroperitoneal sarcoma, colorectal, and stomach, all of which were treated by more than 50% of graduates (Table 3). Pre-2013 graduates spent significantly more time treating breast cancer (21.1 vs. 9.6%, *p* = 0.046), whereas the post-2013 graduates dedicated more time toward pancreas, hepatopancreatobiliary, and stomach cancer (all *p* < 0.05). Among all respondents, the median number of disease sites treated was 5, and this did not differ between the cohorts (Figure 2). Although most practiced broadly, 36% dedicated > 50% of their clinical time towards a single disease site, most commonly breast (n = 5). Counted among this group, 14% of respondents indicated that they treated only a single disease site. In total, 39% of respondents dedicated at least some time towards general surgery. When asked how the clinical scope of their practice had changed over time, 48.8% indicated their practice had become more focused, 26.7% indicated no significant change, 8.9% reported a broader practice with time, and 15.6% reported a change in clinical focus without a significant change in breadth.

**Table 2:** Percentage of respondents’ time spent treating various disease sites and on non-clinical activities

	Average % dedicated time	Pre-2013 graduates	Post-2013 graduates	*p* value
Current clinical practice				
Breast	15.8	21.1	9.6	**0.046**
Melanoma	13.4	13.8	12.9	0.351
Pancreas	7.6	3.3	12.8	**0.008**
Peritoneal surface malignancy	7.4	5.6	9.5	0.183
Colon and rectum	7.3	5.9	9.0	0.374
Hepatobiliary	6.3	2.5	10.8	**0.004**
Retroperitoneal sarcoma	4.7	3.6	5.9	0.931
Stomach	4.6	2.5	7.0	0.034
General surgery (non-oncologic)	3.0	3.2	2.7	0.674
Extremity sarcoma	2.9	2.8	3.1	0.382
Thyroid/parathyroid	2.8	4.3	1.0	0.196
Urologic oncology	2.1	3.9	0.0	0.192
General non-oncologic surgery in patients with cancer	1.6	1.9	1.3	0.894
Esophagus	1.4	1.6	1.2	0.687
Other head and neck	0.6	0.8	0.5	0.262
Gynecologic oncology	0.6	0.2	1.2	0.384
Thoracic (excluding esophagus)	0.0	0.1	0.0	0.361
Non-clinical activities				
Leadership/administration	8.9	15.3	1.3	**0.004**
Clinical research	4.2	5.5	2.7	0.511
Surgical education	2.7	2.0	3.6	0.381
Basic science research	1.6	0.0	3.5	0.052
Public health	0.5	0.3	0.8	0.454

Bold indicates a significant *p*-value < 0.05

Each respondent’s total time was required to add to 100%.

**Table 3:** Percentage of respondents who indicated that they dedicated any amount of time towards the treatment of a particular disease site or were involved in a non-clinical activity

	% treating disease site	Pre-2013 graduates	Post-2013 graduates	*p* value
Current clinical practice				
Melanoma	75.0	62.5	90.0	**0.036**
Retroperitoneal sarcoma	54.5	54.2	55.0	0.956
Colon and rectum	52.3	50.0	55.0	0.741
Stomach	50.0	37.5	65.0	0.069
Breast	47.7	62.5	30.0	**0.032**
Pancreas	45.5	29.2	65.0	**0.017**
Hepatobiliary	45.5	29.2	65.0	**0.017**
Extremity sarcoma	43.2	50.0	35.0	0.317
Peritoneal surface malignancy	38.6	29.2	50.0	0.158
General surgery (non-oncologic)	34.1	37.5	30.0	0.601
Thyroid/parathyroid	27.3	33.3	20.0	0.323
General non-oncologic surgery in patients with cancer	20.5	20.8	20.0	0.946
Esophagus	18.2	20.8	15.0	0.617
Other head and neck	18.2	25.0	10.0	0.199
Gynecologic oncology	15.9	12.5	20.0	0.498
Urologic oncology	4.5	8.3	0.0	0.186
Thoracic (excluding esophagus)	2.3	4.2	0.0	0.356
Non-clinical activity				
Clinical research	45.5	45.8	45.0	0.956
Leadership/administration	40.9	58.3	20.0	**0.010**
Surgical education	36.4	33.3	40.0	0.647
Public health	11.4	8.3	15.0	0.488
Basic science research	6.8	0.0	15.0	**0.049**

Bold indicates a significant *p*-value < 0.05

We also examined how gender affected the current practice of respondents. There were 13 female and 31 male graduates who completed this question. When compared with male surgeons, female surgeons dedicated on average less time towards the treatment of pancreatic, colorectal, hepatobiliary, gastric, and thyroid/parathyroid diseases. In addition, female surgeons dedicated less time to general, non-oncologic surgery in patients with cancer. Female surgeons treated a median of two compared with a median of six disease sites for male surgeons (*p* = 0.001). Additionally, female surgeons tended to be less likely to dedicate time towards general surgery than were male surgeons, with 15.4% of female and 48.4% of male surgeons spending any amount of time on general surgery (*p* = 0.050). There were no significant differences between male and female surgeons in the amount of time dedicated to non-clinical activities.

Respondents were asked the degree to which they had integrated robotic surgery into their practice, using a scale from 1 to 5, with 1 indicating not at all, 3 indicating to a moderate degree, and 5 indicating to a significant degree. The average response was 2.17 for pre-2013 graduates and 3.15 for the post-2013 group (*p* = 0.04). One-quarter of graduates had integrated robotic surgery into their practice to a significant degree, and 63.6% were performing robotic surgery to some extent. In the follow-up survey, respondents were asked, for a variety of surgeries, whether they never, rarely, sometimes, or usually approached the operation robotically. The procedures that were most likely to be approached robotically were minor hepatectomy, distal pancreatectomy, and rectal resection, all of which were “usually” performed robotically in > 40% of cases.

On average, pre-2013 graduates spent slightly more time on non-clinical activities than the later cohort, but this difference was not statistically significant (23 vs. 12%, *p* = 0.093). The earlier cohort dedicated significantly more time towards leadership or administrative activities (15.3 vs. 1.3%, *p* = 0.004). Respondents were asked how their non-clinical activities had evolved over the course of their career. When asked about research, 37.7% had less time for research, 24.4% reported no change, 28.9% now dedicated more time towards research, and 8.9% indicated that they did not participate in research. When asked about leadership, 8.9% had fewer leadership roles, 24.4% had no significant change, and 66.7% devoted more time towards leadership. Finally, 17.3% now had less educational responsibility than when they started, 26.1% reported no significant change, 52.2% indicated more educational responsibility, and 4.3% did not participate in surgical education.

The follow-up survey revealed that respondents who were engaged in leadership were an average of 4.8 years away from completion of fellowship before assuming a leadership role and had spent an average of 7.5 years in leadership or administration. In total, 61% (14/23) had not had any additional formal leadership training. Respondents who were involved in surgical education indicated that they assumed their educational role an average of 1.2 years after graduation from fellowship and had, on average, been involved in education for 12.7 years. Again, the majority (79%, 15/19) had not had any additional formal educational training.

## Discussion

For as long as physicians have treated cancer, the fields of oncology and surgery have been inextricably linked.^1^ Indeed, for most of the past 150 years, surgical extirpation was the only effective treatment available, offering to this day the best likelihood of cure in many cases. General surgeons have performed oncologic operations as part of their broader practice, but even before the term “surgical oncology” existed, there were surgeons whose practice was exclusively devoted to the treatment of cancer. These early cancer surgeons operated broadly, performing radical head and neck surgery, pelvic exenteration for gynecologic malignancies, and major amputations of tumors of the extremities. They also frequently delivered radiation therapy, available since the beginning of the twentieth century, and chemotherapy, which arrived later in the 1940s and 1950s.^1^ Training in surgical oncology has also existed since the 1930s but was formalized in the 1980s, initially under the direction of the SSO and later the ACGME.^3,4^ Just as the surgical oncologist of the 1980s was much different from those at the turn of the century, so are today’s surgical oncologists different from those who came before. As the field of surgical oncology evolves, fellowship training must evolve to prepare graduates for the needs of the field as it exists today.

Our survey provides insights into the practice of surgical oncologists today as well as the evolution of surgical oncology training and practice at our fellowship over the last 4 decades. Broadly, our findings are concordant with those of previous surveys of graduates of surgical oncology training.^8,11,12^ However, by capitalizing on the experiences of surgical oncology surgeons trained over 40 years, we report several interesting trends that emerged from our data regarding the changing breadth and scope of surgical oncology, the growth of robotic surgery, and the involvement of surgical oncologists in leadership and administration.

That the scope of surgical oncology is changing is undeniable. Coincident with the general trend in medicine and surgery towards subspecialization,^13^ the breadth of conditions treated by surgical oncologists has narrowed with time. This is best evidenced by our data comparing the preparedness for practice in pre-2013 and post-2013 graduates. The later cohort felt less well prepared to treat melanoma, stomach, endocrine, extremity sarcoma, head and neck, esophagus, and thoracic malignancies. Melanoma and gastric cancer are both fundamental diseases treated by surgical oncologists, but other areas such as extremity sarcoma may also be treated by oncologic orthopedic surgeons, and still others—including head and neck cancer and thoracic malignancies—are, depending on location, primarily managed by other surgical specialties. This is further supported by our data on the current practice of graduates, with a minority dedicating any time towards esophagus, head and neck, or thoracic oncology. At the same time, the later graduates felt significantly better prepared to treat peritoneal surface malignancies, which represent a growing field within surgical oncology. That the post-2013 graduates also reported feeling better prepared to treat gynecologic malignancies likely represents an idiosyncrasy of our institution. Until recently, our institution did not have a separate gynecologic oncology fellowship, and this resulted in the surgical oncology fellows working closely with the gynecology faculty.

However, our data do not support the idea that all or even most surgical oncologists are becoming super subspecialized within the field. Most respondents treated a wide variety of malignancies, with a median of five oncologic disease sites being treated, and only 36% dedicating >50% of their clinical time to a single disease site. Additionally, 39% of respondents dedicated some time toward general surgery. This breadth of practice was seen in respondents across all practice settings, from academic to community, and is consistent with the literature.^8,12^ These findings suggest that there remains a need to train for broad-based general surgical oncologists since such training will likely prepare graduates well for their eventual employment. Training the super specialized surgeon-scientists who advance the field is a laudable goal of fellowship training, but so too is the preparation of more clinically focused, general oncologic surgeons who meet the need for quality cancer care where it exists. When one considers that roughly 80% of cancer care in the United States is delivered in the community setting rather than at a National Cancer Institute designated center,^14,15^ the importance of training surgeons who can operate outside of academia is obvious. Still, very few respondents treated the entire range of conditions trained in fellowship, which suggests there may be an opportunity to tailor fellowship training by increasing the range of electives directed at specific areas of interest, especially during the second year of training as career goals solidify.

Although the pre-2013 and post-2013 cohorts did not differ significantly in the number of disease sites treated, there were some differences in the practices of the early and later groups. The post-2013 graduates reported dedicating significantly more time toward the treatment of pancreas, hepatobiliary, and gastric cancer, and the pre-2013 graduates devoted more time towards breast cancer. That the older cohort was more likely to treat breast cancer is unsurprising. Separate breast surgery fellowships were established and proliferated during the time period studied, both at our institution and across the country. It is likely that surgical trainees with a strong interest in breast surgery are now less inclined to pursue broad-based surgical oncology training when a more focused alternative exists. In addition, respondents most frequently indicated that, over time, their practice became more clinically focused, involving fewer disease sites.

The changing landscape of surgical oncology is also evident in our data regarding robotic surgery. Currently, most general surgical oncology procedures nationwide are performed open or laparoscopically. However, this is rapidly evolving with the increasing adoption of the robotic platform for surgery of the esophagus, stomach, liver, pancreas, small bowel, colon, and rectum.^16,17^ In our own data, those who graduated before 2004 were inadequately prepared by fellowship to perform robotic surgery, whereas those who graduated after 2018 all reported feeling well prepared. There was a transitional period from 2004 to 2017, as shown in Fig. 1. Furthermore, just over 60% of our graduates, including many who did not train on the robot during fellowship, were currently performing robotic surgery in their practice. As robotic platforms continue to improve, along with the literature supporting their use, adoption and penetrance should only be expected to increase. It is vital that CGSO fellows are well trained in the latest surgical techniques, both to provide the best patient care and to remain competitive in the evolving job market.

Finally, we find that the pre-2013 graduates, who are later in their careers, spend significantly more time in leadership or administrative roles. This finding is not unexpected, but what is noteworthy is the high numbers of respondents in leadership roles (40.9% of all respondents and 58.3% of the pre-2013 cohort). When considered alongside clinical disease sites, the pre-2013 group dedicated an average of 15.3% of their time towards leadership or administration, more than any single disease site other than breast cancer. Although there likely is a trend towards increasing leadership involvement with time in any medical specialty, we contend that the multidisciplinary focus and clinical breadth of surgical oncology training uniquely position graduates to serve as leaders, as supported by studies showing that surgical oncologists are relatively overrepresented as chairs of surgery.^18,19^ Currently, formalized leadership training is not part of the ACGME CGSO curriculum nor part of the training at our fellowship. However, when asked if there were any areas that respondents would have liked additional exposure to in fellowship, the most commonly selected answer was leadership. The form this leadership training might assume remains uncertain. Further investigation in this topic is warranted if graduates are likely to assume leadership roles during their career.

Other non-clinical activities, including surgical education, public health, and research occupied significantly less of respondents’ time. Although surgical oncology fellowship currently includes 4 months of research, the dedicated research time is insufficient to seriously prepare graduates for a career in research and may detract from clinical training in those who do not plan to pursue research. Among our respondents, 45% were involved in clinical research, and only 7% dedicated time towards basic science research, findings consistent with prior surveys.^8,12^ Familiarity with fundamental research and statistical techniques is essential for a critical understanding of the surgical oncology literature. However, for most fellows it is likely that this foundation of knowledge has been built during dedicated research time in residency. For the other half of fellows who will not dedicate significant or any time towards research, it may be more useful to spend the 4 months currently allocated to research on clinical electives. Similarly, for those who are serious about embarking on a research career, the opportunity to spend even more time developing these skills would be beneficial.

A major strength of the present study is the 40-year time period covered, which allowed us to draw conclusions about the evolution of surgical oncology training and the practice of surgical oncologists. Additionally, the granularity with which respondents were asked to assess their readiness and describe their current practice allows for a finer understanding of the field. Limitations of the present study include those inherent to survey studies. These include non-response bias (45% non-response rate) and potential for recall bias. Additionally, as a single-institute study, the results may not be generalizable to other fellowship programs. Our survey results highlight ways in which our own program’s training has successfully evolved to meet the changing practice of surgical oncology, for instance with the introduction of robotic training and relative de-emphasis of thoracic and head and neck surgery. At the same time, we can identify trends in training that do not reflect an actual change in graduates’ practices, such as the later cohort feeling relatively less prepared to treat melanoma. Similar studies performed at other institutions may improve on our understanding of surgical oncology training across the different sites, recognize common themes across multiple programs, and identify the unique strengths or gaps in individual programs, whereas studies such as those by Ruff et al. and Beherns et al. using the broader cohort of CGSO graduates can help identify trends on a national level.^8,12^

## Conclusions

In this survey of graduates from a single surgical oncology fellowship between 1981 and 2022, we found that surgical oncology fellowship and practice were generally well aligned, and that fellows felt well prepared to treat a wide range of malignancies. During this time, the scope of training narrowed, with a decreased emphasis on esophageal, thoracic, and head and neck cancers, mirroring the realities of the respondents’ current practice. Respondents tended to have a varied but not all-encompassing practice. Such practice patterns support the current broad-based training paradigm but also suggest that there are opportunities to tailor training to individual fellows’ goals. As their careers progressed, respondents dedicated more time towards leadership, with nearly 60% of those who graduated before 2013 involved in leadership or administration. Some form of leadership training in fellowship may better prepare graduates for leadership positions.

## Supplementary Information

Below is the link to the electronic supplementary material.

Supplementary file1 (DOCX 42 KB)

Supplementary file2 (DOCX 41 KB)

## References

[1] Lawrence W, et al. History of surgical oncology. In: JA Norton, PS Barie, RR Bollinger, et al., editors. Surgery: basic science and clinical evidence. New York: Springer; 2008. p. 1889–900.

[2] Blazer DG 3rd. Supply and demand: is the surgical oncology match in a bear market? *Ann Surg Oncol*. 2022;29(13):7947–9. 10.1245/s10434-022-12544-7. 36103015 10.1245/s10434-022-12544-7 PMC9472720

[3] Schweitzer RJ, Edwards MH, Lawrence W Jr, Mozden PJ, Scanlon EF, Leffall LD Jr. Training guidelines for surgical oncology. *Cancer*. 1981;48(10):2336–40. 10.1002/1097-0142(19811115)48:10<2336::aid-cncr2820481033>3.0.co;2-t. 7296484 10.1002/1097-0142(19811115)48:10<2336::aid-cncr2820481033>3.0.co;2-t

[4] Michelassi F. 2010 SSO presidential address: subspecialty certificate in advanced surgical oncology. *Ann Surg Oncol*. 2010;17(12):3094–103. 10.1245/s10434-010-1286-7. 20803078 10.1245/s10434-010-1286-7

[5] Balch CM, Coit DG, Berman RS. 2015 James Ewing lecture: 75-year history of the society of surgical oncology-part III: the transformative years (1991–2015). *Ann Surg Oncol*. 2016;23(5):1409–17. 10.1245/s10434-015-5011-4. 26822879 10.1245/s10434-015-5011-4

[6] Silvestre J, Smith JR, Nasef KE, Wilson LL, Kelz RR. Application and match rates in the complex general surgical oncology match. *Ann Surg Oncol*. 2022;29(13):8094–8. 10.1245/s10434-022-12428-w. 35999416 10.1245/s10434-022-12428-w PMC9398039

[7] Wach MM, Ruff SM, Ayabe RI, et al. An examination of applicants and factors associated with matriculation to complex general surgical oncology fellowship training programs. *Ann Surg Oncol*. 2018;25(12):3436–42. 10.1245/s10434-018-6674-4. 30054823 10.1245/s10434-018-6674-4 PMC6191290

[8] Behrens S, Lillemoe HA, Dineen SP, et al. Perceptions of readiness for practice after complex general surgical oncology fellowship: a survey study. *Ann Surg Oncol*. 2024;31(1):31–41. 10.1245/s10434-023-14524-x. 37936022 10.1245/s10434-023-14524-x PMC10695882

[9] Keswani SG, Moles CM, Morowitz M, et al. The future of basic science in academic surgery: identifying barriers to success for surgeon-scientists. *Ann Surg*. 2017;265(6):1053–9. 10.1097/SLA.0000000000002009. 27643928 10.1097/SLA.0000000000002009 PMC5450912

[10] Alverdy JC. Surgeon as basic bench scientist: a play in three acts. *J Surg Res*. 2019;241:336–42. 10.1016/j.jss.2019.04.016. 31071482 10.1016/j.jss.2019.04.016 PMC9382856

[11] Heslin MJ, Coit DG, Brennan MF. Surgical oncology fellowship: viable pathway to academic surgery? *Ann Surg Oncol*. 1999;6(6):542–5. 10.1007/pl00021735. 10493621 10.1007/pl00021735

[12] Ruff S, Ilyas S, Steinberg SM, et al. Survey of surgical oncology fellowship graduates 2005–2016: insight into initial practice. *Ann Surg Oncol*. 2019;26(6):1622–8. 10.1245/s10434-019-07220-2. 30761439 10.1245/s10434-019-07220-2 PMC8170836

[13] Bruns SD, Davis BR, Demirjian AN, et al. The subspecialization of surgery: a paradigm shift. *J Gastrointest Surg*. 2014;18(8):1523–31. 10.1007/s11605-014-2514-4. 24756925 10.1007/s11605-014-2514-4

[14] Bilimoria KY, Stewart AK, Winchester DP, Ko CY. The national cancer data base: a powerful initiative to improve cancer care in the United States. *Ann Surg Oncol*. 2008;15(3):683–90. 10.1245/s10434-007-9747-3. 18183467 10.1245/s10434-007-9747-3 PMC2234447

[15] Fong ZV, Chang DC, Hur C, et al. Variation in long-term oncologic outcomes by type of cancer center accreditation: an analysis of a SEER-medicare population with pancreatic cancer. *Am J Surg*. 2020;220(1):29–34. 10.1016/j.amjsurg.2020.03.035. 32265013 10.1016/j.amjsurg.2020.03.035 PMC8350560

[16] Stewart CL, Ituarte PHG, Melstrom KA, et al. Robotic surgery trends in general surgical oncology from the national inpatient sample. *Surg Endosc*. 2019;33(8):2591–601. 10.1007/s00464-018-6554-9. 30357525 10.1007/s00464-018-6554-9

[17] Hays SB, Corvino G, Lorie BD, et al. Prince and princesses: the current status of robotic surgery in surgical oncology. *J Surg Oncol*. 2024;129(1):164–82. 10.1002/jso.27536. 38031870 10.1002/jso.27536

[18] Tanious A, McMullin H, Jokisch C, et al. Defining a leader-characteristics that distinguish a chair of surgery. *J Surg Res*. 2019;242:332–5. 10.1016/j.jss.2019.04.082. 31129242 10.1016/j.jss.2019.04.082

[19] Slakey DP, Korndorffer JR, Long KN, Clark T, Hidalgo M. The modern surgery department chairman: the job description as identified by chairmen. *JAMA Surg*. 2013;148(6):511–5. 10.1001/jamasurg.2013.1230. 23754568 10.1001/jamasurg.2013.1230

