# Supplementary material for: Practice Patterns of Graduates from a Surgical Oncology Fellowship Program
Source: Ann Surg Oncol. 2026 Jan 22;33(6):4940–8. doi: 10.1245/s10434-025-19073-z (PMC13179182; doi:10.1245/s10434-025-19073-z)
Supplement: Supplementary file 1 — Supplementary file1 (DOCX 42 KB) [file 10434_2025_19073_MOESM1_ESM.docx]

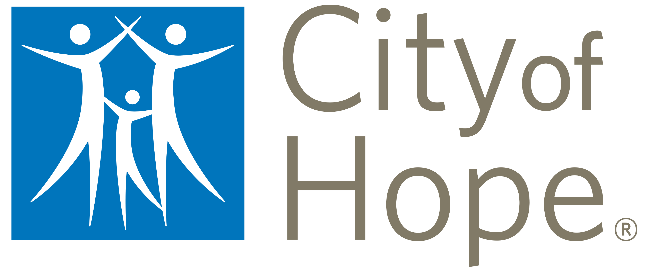


| **Practice Patterns of City of Hope Surgical Oncology Alumni** |
| --- |

**Demographics**

1. What is your gender?

Male

Female

Other

Prefer not to answer

2. At what institution did you complete your general surgery residency

3. In what year did you finish fellowship training?

4. Are you board certified in complex general surgical oncology?

Yes

No

|  |
| --- |

**Fellowship Training**

Top of Form

5. Prior to starting fellowship, did you have a specific area of clinical interest?

6. How well did completion of a surgical oncology fellowship prepare you to manage the following disease sites? (Rated on 1 – 5 scale. 1: inadequately prepared, 3: adequately prepared, 5: very well prepared)

Thyroid/parathyroid

Other head and neck

Breast

Melanoma

Thoracic oncology (excluding esophagus)

Esophagus

Stomach

Pancreas

Hepatobiliary

Colon and rectum

Peritoneal surface malignancy

Retroperitoneal sarcoma

Extremity sarcoma

Gynecologic oncology

Urologic oncology

Non-oncologic surgery in cancer patients

7. How well did completion of a surgical oncology fellowship prepare you for the following non-clinical activities? (Rated on 1 – 5 scale. 1: inadequately prepared, 3: adequately prepared, 5: very well prepared)

Basic science research

Clinical research

Public health research

Leadership/administration

Surgical education

8. Are there any areas that you would have liked to have had more exposure to during fellowship?

Thyroid/parathyroid

Other head and neck

Breast

Melanoma

Thoracic (excluding esophagus)

Esophagus

Stomach

Pancreas

Hepatobiliary

Colon and rectum

Peritoneal surface malignancy

Retroperitoneal sarcoma

Extremity sarcoma

Gynecologic oncology

Urologic oncology

Non-oncologic surgery in cancer patients

Basic science research

Clinical research

Public health

Leadership/administration

Surgical education

Other (please specify)

9. Are there any areas of training that you felt were not useful, or were overemphasized during fellowship?

Thyroid/parathyroid

Other head and neck

Breast

Melanoma

Thoracic (excluding esophagus)

Esophagus

Stomach

Pancreas

Hepatobiliary

Colon and rectum

Peritoneal surface malignancy

Retroperitoneal sarcoma

Extremity sarcoma

Gynecologic oncology

Urologic oncology

Non-oncologic surgery in cancer patients

Basic science research

Clinical research

Public health

Leadership/administration

Surgical education

Other (please specify)

10. To what extent did your fellowship training prepare you to perform robotic surgery? (Rated on 1 – 5 scale. 1: inadequately prepared, 3: adequately prepared, 5: very well prepared)

11. Do you have any additional subspecialty training?

No

Yes - Please specify

12. If you answered yes to the above, was the additional training obtained before or after fellowship?

Before fellowship

After fellowship

13. Given the opportunity to choose again, would you change your decision to complete a surgical oncology fellowship?

No

Yes - Alternative subspecialty training

Yes - General surgery practice

Yes - Research/Industry/Other - Please specify

**Post Fellowship Practice**

14. What is your current practice setting?

University/academic hospital

Freestanding cancer center

Academic affiliate/hybrid practice

Private practice

Other (please specify)

15. What is the location of your current practice?

16. How long have you been at your current practice?

17. Including your current practice, how many jobs have you had since fellowship?

18. How many partners do you have in your current practice?

19. During the last year, what percentage of your time has been allocated to the following areas? (Answers must add up to 100)

Thyroid/parathyroid

Other head and neck

Breast

Melanoma

Thoracic oncology (excluding esophagus)

Esophagus

Stomach

Pancreas

Hepatobiliary

Colon and rectum

Peritoneal surface malignancy

Retroperitoneal sarcoma

Extremity sarcoma

Gynecologic oncology

Urologic oncology

General surgery (non-oncologic)

General non-oncologic surgery in cancer patients

Basic science research

Clinical research

Public health

Leadership/administration

Surgical education

20. To what degree have you integrated robotic surgery into your surgical practice?

21. How has the breadth of your clinical practice changed since you took your first job?

More focused (fewer diseases treated)

No significant change

Change in clinical focus without change in breadth

Broader (more diseases treated)

22. How has your research focus changed since you took your fist job?

Less time dedicated to research

No change in research time

More time dedicated to research

N/A - Do not participate in research

23. How has your time spent in leadership or administrative roles changed since you took your first job?

Fewer leadership roles

No significant change

More leadership roles

NA - No leadership roles

24. How has your educational responsibility changed since you took your first job?

Less educational responsibility

No significant change

More educational responsibility

NA - No educational responsibility

Bottom of Form
