# Supplementary material for: Practice Patterns of Graduates from a Surgical Oncology Fellowship Program
Source: Ann Surg Oncol. 2026 Jan 22;33(6):4940–8. doi: 10.1245/s10434-025-19073-z (PMC13179182; doi:10.1245/s10434-025-19073-z)
Supplement: Supplementary file 2 — Supplementary file2 (DOCX 41 KB) [file 10434_2025_19073_MOESM2_ESM.docx]

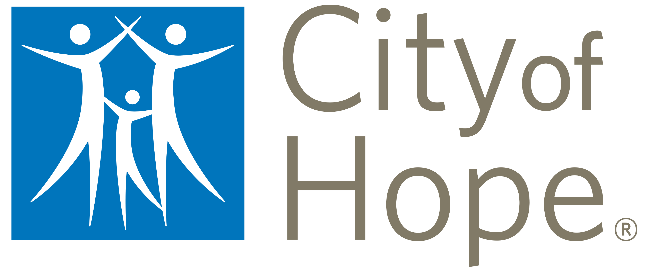


| **Practice Patterns of City of Hope Graduates - Follow-up Survey** |
| --- |

Demographic Data

Top of Form

1. What is your gender?

Male

Female

Prefer not to answer

2. Where did you complete your general surgery residency?

3. In what year did you complete your general surgery residency?

**Bottom of Form**

**Leadership/Administration**

For respondents who indicated that they dedicated time to leadership/administration, please answer the following questions.

Top of Form

4. What, if any, is the official title of your leadership position?

5. For how many years have you been involved in leadership/administration?

6. How many years from fellowship were you before assuming a leadership role?

7. Did you complete any additional leadership or administrative training? If so, please specify.

8. Did your additional training lead to a degree?

9. What, if any, leadership or administrative training do you think should be incorporated into surgical oncology fellowship?

**Education**

For respondents who indicated that they dedicated time to education, please answer the following questions.

10. What, if any, is the official title of your educational position? If no formal title, please respond with “none.” If multiple educational positions are held, please list each.

11. For how many years have you been involved in education?

12. How many years out from fellowship were you before assuming an educational role?

13. Did you complete any additional training in medical education? If so, please specify.

14. Did your additional training lead to a degree?

15. What, if any, additional medical education training do you think should be incorporated into surgical oncology fellowship?

**Robotic Surgery**

Top of Form

16. For those who have not integrated robotic surgery into their practice, or have done so to a limited degree, what factors have prevented you from doing so? If multiple factors contribute, please select all that apply.

Robotic approach is not applicable to the disease sites treated.

Lack of clear benefit to robotic approach for disease sites treated.

Lack of familiarity or training with the robotic system.

Limited or no access to the robotic system.

Other (please specify)

17. For those who have not integrated robotic surgery into their practice, do you intend to do so in the future?

Yes

No

18. For those who have integrated robotic surgery into their practice, how important was robotic training/certification/competency in obtaining your current job?

19. For each of the following procedures please indicate how frequently you perform the procedure robotically. (For each procedure, respondents chose one of the following: Usually, sometimes, rarely, never, n/a do not perform this procedure)

Lung resection

Esophagectomy

Gastrectomy

Cholecystectomy (benign disease)

Cholecystectomy (for malignancy)

Pancreaticoduodenectomy

Distal pancreatectomy

Pancreatic enucleation

Splenectomy

Major hepatectomy (4 or more segments)

Minor hepatectomy

Colectomy

Rectal resection

Transanal resection

Small bowel resection

Adrenalectomy

Retroperitoneal/Abdominal lymphadenectomy or lymph node biopsy

Hysterectomy

Oophorectomy

Nephrectomy

Cystectomy

Prostatectomy

Diagnostic laparoscopy

Anti-reflux surgery

Ventral hernia repair

Inguinal hernia repair

Feeding access (gastrostomy, jejunostomy)Bottom of Form

Bottom of Form
